# Culturally adapted psychosocial interventions (CaPSI) for early psychosis in a low-resource setting: study protocol for a large multi-center RCT

**DOI:** 10.1186/s12888-023-04904-8

**Published:** 2023-06-16

**Authors:** M. O. Husain, A. B. Khoso, T. Kiran, N. Chaudhry, M. I. Husain, M. Asif, M. Ansari, A. H. Rajput, S. Dawood, H. A. Naqvi, A. T. Nizami, Z. Tareen, J. Rumi, S. Sherzad, H. A. Khan, M. R. Bhatia, K. M. S. Siddiqui, Z. Zadeh, N. Mehmood, U. Talib, C. de Oliveira, F. Naeem, W. Wang, A. Voineskos, N. Husain, G. Foussias, I. B. Chaudhry

**Affiliations:** 1 Campbell Family Mental Health Research Institute, Centre for Addiction and Mental Health, 1025 Queen St West, Toronto, ON Canada; 2 Department of Psychiatry, Temerty Faculty of Medicine, University of Toronto, Toronto, Canada; 3 Pakistan Institute of Living and Learning, Karachi, Pakistan; 4 Department of Psychiatry, Liaquat University of Medical and Health Sciences, Hyderabad, Pakistan; 5 Centre for Clinical Psychology, University of the Punjab, Lahore, Pakistan; 6 Department of Psychiatry, Dow University Health Sciences, Karachi, Pakistan; 7 Institute of Psychiatry, Benazir Bhutto Hospital, Rawalpindi, Pakistan; 8 Department of Psychiatry, Balochistan Institute of Psychiatry & Behavioural Sciences, Quetta, Pakistan; 9 Department of Psychiatry, Peoples University of Medical and Health Sciences, Shaheed Benazirabad, Pakistan; 10 National Psychiatric Hospital, Multan, Pakistan; 11 Institute for Mental Health, Karwan-E-Hayat, Karachi, Pakistan; 12 Institute for Mental Health Policy Research, Centre for Addiction and Mental Health, Toronto, ON Canada; 13 Institute of Health Policy, Management and Evaluation, Dalla Lana School of Public Health, University of Toronto, Toronto, Canada; 14 ICES, Toronto, Canada; 15 Mersey Care NHS Foundation Trust, Prescott, UK; 16 Division of Psychology and Mental Health, University of Manchester, Manchester, UK; 17 Department of Psychiatry, Ziauddin University, Karachi, Pakistan

**Keywords:** Early psychosis, Psychosocial interventions, Randomized control trial, Pakistan

## Abstract

**Background:**

Psychosis treatment guidelines recommend cognitive behaviour therapy (CBT) and family intervention (FI), for all patients with first episode psychosis (FEP), though guidance borrows heavily from literature in adults from high income countries. To our knowledge, there are few randomized controlled trials (RCTs) examining the comparative effect of these commonly endorsed psychosocial interventions in individuals with early psychosis from high-income countries and no such trials from low and middle-income countries (LMICs). The present study aims to confirm the clinical-efficacy and cost-effectiveness of delivering culturally adapted CBT (CaCBT) and culturally adapted FI (CulFI) to individuals with FEP in Pakistan.

**Method:**

A multi-centre, three-arm RCT of CaCBT, CulFI, and treatment as usual (TAU) for individuals with FEP (*n* = 390), recruited from major centres across Pakistan. Reducing overall symptoms of FEP will be the primary outcome. Additional aims will include improving patient and carer outcomes and estimating the economic impact of delivering culturally appropriate psychosocial interventions in low-resource settings. This trial will assess the clinical-efficacy and cost-effectiveness of CaCBT and CulFI compared with TAU in improving patient (positive and negative symptoms of psychosis, general psychopathology, depressive symptoms, quality of life, cognition, general functioning, and insight) and carer related outcomes (carer experience, wellbeing, illness attitudes and symptoms of depression and anxiety).

**Conclusions:**

A successful trial may inform the rapid scale up of these interventions not only in Pakistan but other low-resource settings, to improve clinical outcomes, social and occupational functioning, and quality of life in South Asian and other minority groups with FEP.

**Trial registration:**

NCT05814913.

**Supplementary Information:**

The online version contains supplementary material available at 10.1186/s12888-023-04904-8.

## Background

Psychosis is one of the 20 leading causes of disability worldwide, affecting 29 million people [1]. A large burden of disease is attributed to people in low and middle-income countries (LMICs) [2]. First Episode Psychosis (FEP) occurs at a young age and is a critical period influencing the long-term course of the disorder. Early psychosis is characterized by repeated relapses, with up to 80% relapsing within 5 years of an initial episode [3]. Relapse causes distress as well as disruption of social and occupational functioning. Relapse following FEP affects long-term psychosocial development at pivotal developmental stages. During early psychosis, pharmacological, social and cognitive interventions can profoundly impact long-term functional outcomes [4]. Antipsychotic medication is effective in reducing risk of relapse [3], however, a significant proportion of patients continue to experience distressing symptoms despite adherence to medication [4].

### Psychosocial interventions for psychosis

Although psychosis treatment guidelines [5–7] endorse psychosocial interventions, namely cognitive behavior therapy (CBT) and family intervention (FI), for all patients with FEP, there are few clinical efficacy trials examining the comparative effects of these interventions in FEP from high-income countries and no such trials to our knowledge from LMICs. The last two decades have seen advances in the development of effective non-pharmacological treatments for psychosis including CBT and FI. A 2021 meta-analysis identified FI and CBT among the most efficacious psychosocial interventions to prevent psychosis relapse in schizophrenia [8]. Meta-analyses have demonstrated that CBT is effective in improving positive and negative symptoms [9], adherence to medication, coping strategies, insight, quality of life and functioning in psychosis [10, 11]. Few studies have shown CBT alone to be more effective than routine care in patients with FEP [12], but use of CBT as an adjunct to pharmacotherapy has been endorsed internationally [5, 13]. Family support is particularly important for those experiencing FEP as illness onset typically occurs when patients are living with caregivers [14]. FEP can be a challenging time as relatives and carers struggle to come to terms with the illness [15]. In LMICs, the responsibility on family members to provide care is further compounded by the lack of trained mental health workers, insufficient resources, and inadequate infrastructure to support mental health services. Families and carers of individuals with psychosis report significant distress, lower quality of life and increased anxiety and depression [16]. Family interventions for psychosis are recommended internationally and have been shown to significantly reduce relapse and readmission rates [17], improve medication adherence [18], enhance functioning [19] and improve family environment [20]. There is evidence for the positive contribution of families towards the wellbeing of people with psychosis, especially when family members are actively supported by psychoeducation, a core component of FI [18, 21]. However, there is limited evidence from LMICs that supports the clinical efficacy and cost-effectiveness of delivering psychosocial interventions in FEP [22, 23].

CBT and FI, like many other modern therapies, were first developed in the West, and as such, largely represent Western cultural values [24]. Social, religious, and cultural factors are known to influence the perception of mental illness, in turn impacting health-related behaviour and engagement with services. The need to culturally adapt these interventions before applying them in non-Western LMICs is clear. Our team has led the first pilot randomized controlled trials (RCTs) of culturally adapted CBT (CaCBT) and culturally adapted FI (CulFI) added to treatment as usual (TAU) for patients with schizophrenia in Pakistan [25, 26]. Building on the preliminary efficacy demonstrated by these pilot RCTs, we propose a three-arm RCT comparing a CBT-focused vs. family-focused intervention vs. TAU for people with psychosis, in a LMIC setting, with the ultimate intent of informing scalable evidence-based care.

### Study objectives

1. To determine the clinical efficacy of CaCBT and CulFI compared to TAU on reducing overall symptoms of psychosis in patients with FEP in Pakistan.
2. To determine the efficacy of CaCBT and CulFI compared to TAU on positive and negative symptoms of psychosis, general psychopathology, depressive symptoms, quality of life, general functioning, and insight in patients with FEP in Pakistan.
3. To determine the efficacy of CaCBT and CulFI compared to TAU on improving carer experience, carer wellbeing, carer illness attitudes and symptoms of depression and anxiety in family and carers of patients with FEP in Pakistan.
4. To determine the comparative effect of CaCBT and CulFI in improving patient and carer-related outcomes in individuals with FEP in Pakistan.
5. To estimate the cost-effectiveness of delivering culturally appropriate psychosocial interventions in low-resource settings.
6. To explore delivery and reach of each intervention, tolerability of intervention components, acceptability of interventions, understanding mechanism of change and developing an understanding of barriers and facilitators to future adoption using process evaluation.

## Methods/design

### Study design and setting

**Fig. 1:**
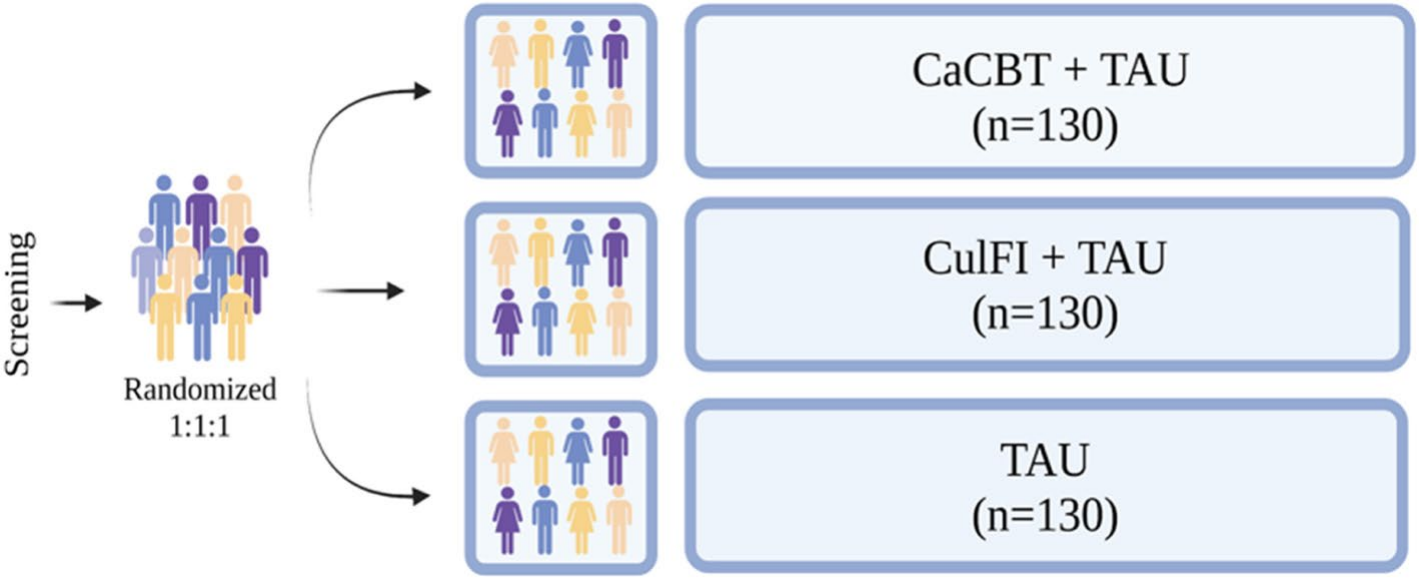
Study Schematic

This is an assessor-masked, three-arm RCT design (trial registration: NCT05814913). Participants (*N* = 390) will include adults with FEP, and will be recruited from ten major recruitment centres (i.e., Karachi, Lahore, Rawalpindi, Hyderabad, Qambar Shahdakot, Shaheed Benazirabad, Sukkur, Peshawar, Quetta and Multan) (*n* = 390, 130 per condition; please see Fig. 1). By recruiting participants from across the country, we are confident that the sample will be representative of Pakistani patients with psychosis.

### Participants

Participants will be included in the study according to the eligibility criteria (Additional file 1: Appendix 1).

### Study interventions

#### Culturally Adapted Cognitive Behaviour Therapy (CaCBT) for psychosis

Participants in the CaCBT group will include adults with FEP who receive 12 individual sessions from a trained Masters level research psychologist over 12-weeks. The number of sessions has been informed by our pilot work [26], where we demonstrated strong recruitment and retention rates, as well as promising effects on improvement of psychosis symptoms. At least 45 min of therapy is offered once a week during the three-month period. Failure to engage will be defined as attendance of less than six therapy sessions (< 50%). Participants will continue TAU alongside the intervention. The CaCBT intervention is based on the intervention manual developed by David Kingdon and Douglas Turkington [27], and culturally adapted by our group. Adaptation focuses on delivery and engagement rather than CBT content. The use of culturally appropriate idioms, drawing on religious teaching, local stories and images has been effective in explaining symptoms and causes of disorders. Patients and their carers in Pakistan use a bio-psycho-spiritual-social model of illness. They seek help from various sources, including faith healers. Therapists, therefore, adjust therapy accordingly. CaCBT aims to take a collaborative approach to gaining an understanding of the symptoms experienced, working towards reducing distress and disability. There are distinct stages, including engagement, the examination of antecedents of the emerging psychotic disorder, the development of normalizing rationale, the treatment of co-morbid anxiety or depression, and collaboratively constructing a case formulation. CaCBT uses specific techniques for positive symptoms of psychosis thereafter. For addressing auditory hallucinations, beliefs about the origin and nature of the experiences(s) are explored using collaborative critical analysis. Strategies such as voice diaries, reattributing the cause of the voices, and development of coping strategies are also employed. Guided discovery and graded homework tasks are used to elucidate delusions. Focusing on specific themes, clarification of neologisms, and thought linkage are some of the techniques used to improve thought disorder. After work on positive symptoms, negative symptoms are targeted using activity scheduling and records of mastery and pleasure in a diary.

#### Culturally adapted Family Intervention (CulFI) for psychosis

Participants in the CulFI group will include adults with FEP and their carers, who will receive 10 sessions of 40–60 min, delivered weekly for the first 8 weeks and fortnightly for the remaining 4 weeks. Sessions are delivered to both patients and their carers, though patient participation in sessions is not necessary. CulFI consists of two integrated manuals, *“Schizophrenia: The Indian scene*” [28] and *“Families of schizophrenic patients: Cognitive behavioural intervention”* [29]. CulFI comprises: family psychoeducation; cognitive-behavioural skills training for stress-management, coping and problem solving; crisis intervention and suicide risk management; relapse prevention; and education and support regarding the family environment, including communication training. The components are designed to facilitate an understanding about psychosis, the emotional impact of the illness on family relationships, to promote more adaptive coping strategies and minimize relapse risk. In our pilot study [25] we demonstrated strong recruitment, retention, and large effect sizes on carer well-being and support. Adaptations to the intervention, akin to those outlined above for CaCBT, followed a rigorous cultural adaptation framework based on our prior work in Pakistan [24, 26, 30].

### Treatment-as-usual (control condition)

There is little if any provision for psychosocial interventions for patients with FEP in Pakistan [31]. Therefore, TAU is essentially the prescription of antipsychotic medication and follow-up appointments with the responsible physician. The medication history of each patient participant will be recorded at visit one. Changes to the participants’ TAU during the study period are permissible, however, responsible physicians will be encouraged to maintain stable TAU and medication will be recorded at each study visit. There is no TAU provided to carers, however, as partners to care they routinely attend follow-up appointments with patients.

### Outcome measures

Primary outcomes will be reduction in symptom severity in FEP as measured by PANSS [32] total score post intervention (three months), six months and twelve months post intervention. All secondary measures including patient and carer outcomes are listed in Additional file 1: Appendix 2.

### Study procedures

Participants will be recruited from ten major centres each with multiple outpatient psychiatric units that provide mental health care to a combined population of over 40 million people. Clinicians will identify potential participants and offer them information about the study. Those who agree will be contacted by the research team, pre-screened, and provided detailed information about the study including procedures protecting privacy and confidentiality. Participants who consent to participate will then undergo a screening assessment based on the eligibility criteria (Additional file 1: Appendix 1), followed by the baseline assessment. The trial manager will confirm eligibility and assign a participant ID number.

### Randomization and masking

Participants (i.e., patients) will be randomised using a secure web-based randomization system. Participants will be allocated to trial groups using permuted block with variable block sizes stratified by site and sex. We have not included additional stratification variables as per best practice in RCTs [33]. We will assess other potential prognostic characteristics (age, duration of illness, gender, sex) in our a priori subgroup analyses. Therapists will contact the participant within 1 week of randomization to inform them about treatment allocation. The time and date of sessions will be set based on the participant’s preference. Participants randomized to the CaCBT group are patients who will receive 12 individual sessions from a trained Masters level research psychologist over 12-weeks. Each session will last for 60 min. Participants in the CulFI group consist of patients and their carers who will receive 10 sessions of 40–60 min, delivered weekly for the first 8 weeks and fortnightly for the remaining 4 weeks. All sessions will be delivered by trained therapists who will receive two months training from senior therapists before starting intervention. The third group will receive TAU. Assessments will be carried out at baseline, months 3, 6, and 12. These scales will be administered in-person and/or via secure videoconference software by trained, blinded research analysts (RAs). This is an assessor masked RCT and due to the nature of the interventions it will be impossible to mask clinicians in participating centres or the participants themselves allocated to the interventions. Participants will be asked before assessments not to reveal any information about treatment to assessors. To avoid unmasking, outcome assessors will be located separately from treatment providers, and we will assign new outcome assessors in cases of unintentional unmasking. Statistical analysis will be partially masked (using treatment group but not what each group is).

### Sample size

With the proposed total sample size of 390 (130 per condition), we have sufficient power (80%) to detect a small to medium treatment effect (Cohen’s D = 0.39) of either CaCBT or CulFI over TAU on PANSS total score at post-treatment (3-month). The selection of effect size has been informed by a meta-analysis of culturally adapted psychosocial interventions [34] and effect sizes generated from meta-analysis of CBT for psychosis [35]. Cohen’s D of 0.39 is also equivalent to a clinically meaningful reduction in PANSS of 6.70 points. The standard deviation, informed by our pilot work, was taken as 17.18 [26]. For sex or gender-based subgroup analysis, the minimum detectable effect sizes to attain sufficient power increase to 0.48 and 0.67 for men and women respectively. Our exploratory analysis will compare the effect of CulFI with CaCBT in improving patient and carer related outcomes. Although this exploratory outcome is not adequately powered for a non-inferiority analysis, it will inform sample size for future comparative clinical trials. The power calculation has considered 20% attrition at post treatment and was based on an end-point analysis. Longitudinal analysis will be more powerful with reduction in measurement error due to repeated measures.

### Statistical analysis

Descriptive analysis will first be conducted for the baseline data to inspect group differences across the three conditions. Patterns of missing outcome data will be examined as a function of recorded baseline characteristics and patterns of compliance with the protocol. For the primary and secondary aims, linear mixed-effects models will be the main analytic strategy and will be conducted under an intention-to-treat framework. A multiple imputation approach will be employed as the missing data method to account for potential bias caused by attrition. Two types of analysis will be conducted to evaluate the treatment effects of CaCBT and CulFI compared to TAU. An endpoint analysis will focus on the pre-post difference at 3 months and a longitudinal analysis will inspect change overtime (baseline, 3 months, and 6 and 12-months post intervention) regarding long-term effect and sustainability. Treatment assignment and its interaction with time will serve as the primary predictors with site, key demographics and baseline outcome measure being controlled as covariates. For moderation analysis concerning biological sex and gender, we will include interaction between treatment assignment and sex and gender in the endpoint analysis and add time to form three-way interaction in the longitudinal analysis. Moderation effects of sex and gender will be assessed in separate models so their effects can be estimated independently. Similar approaches will be applied for secondary outcomes and generalized linear mixed effects models will be in place for binary outcomes. For the exploratory aim to compare effect of CaCBT and CulFI in improving patient and carer related outcomes, we will conduct noninferiority tests to evaluate if the difference between CaCBT and CulFI is within the noninferiority margin for each individual outcome. The noninferiority margins will be determined carefully following well-accepted guidelines [36] and clinical knowledge.

#### Frequency of analysis

There are no planned interim analyses for efficacy. These will only be carried out if requested by the data monitoring committee. All main statistical analyses will be based on the Intention-to-Treat principle. Analysis will take place after full recruitment and follow-up. During the trial, periodic quality checks of data will be carried out by the trial statistician masked to treatment allocation. Once data entry has been completed preliminary data analysis will be carried out whilst maintaining masking to treatment allocation. The trial will be conducted and reported as per CONSORT recommendations for RCTs.

#### Subgroup analysis

Subgroup analysis will be carried out with respect to age, gender, duration of illness, and sex by adding a treatment with covariate interaction into the primary analysis model. Gender and sex will be included in separate models to evaluate independent impact.

#### Cost-effectiveness analysis

We will undertake an economic evaluation of a culturally appropriate psychosocial intervention, where the target population will be adults living in Pakistan with first episode psychosis (FEP) and a carer. In particular, the economic evaluations (i.e., the cost-effectiveness and cost-utility analyses) of the CaCBT and CulFI interventions will be compared to TAU alone. The economic evaluations will be undertaken from the health care sector (i.e., provider and/or third-party payer) and societal perspectives, which is commonly done for economic evaluations undertaken in low- and middle-income countries [37]. The time horizon will be the duration of the trial and the 12-month period after intervention is delivered and, as a result, a discount rate of 5% will be applied, where necessary, in line with the literature [38].

We will quantify the incremental health gains and costs between the three treatment groups. Changes in psychosis symptom severity will be measured using the Positive and Negative Syndrome Scale (PANSS) [32] for the cost-effectiveness analysis, while quality adjusted life years (QALYs) will be assessed using the EQ-5D [39] and the WHO Disability Assessment Schedule (WHODAS) [40] for the cost-utility analysis. Costs will be obtained through a combination of top-down and bottom-up micro-costing approaches. The costs of the intervention will be collected by the team and will include costs associated with staff time, including that of therapists and their training related to intervention delivery, resources and equipment used to deliver the intervention. A bespoke questionnaire will be used to collect data on health care utilization arising due to psychosis and the treatment of patients, other costs to patients (i.e., travel costs) as well as measures of loss of productivity (i.e., absenteeism defined as number of days-off from work due to acute deterioration) over the 12-month period. We will also include the cost of both formal and informal care provided during the trial. We will use standard published sources to obtain unit costs, where available, and supplement these with data from local sources to convert resource use into monetary values. All costs will be adjusted to 2025 Pakistani rupees using appropriate inflators.

For the cost-effectiveness analysis, we will present the results as the cost per psychosis symptom improvement, while for the cost-utility analysis, we will show the results as the cost per quality-adjusted life year gained 12 months after the intervention. Incremental cost-effectiveness ratios will be calculated as the difference in costs between each intervention and the comparator (i.e., treatment as usual alone) groups divided by the difference in benefits/outcomes for economic evaluation. We will also calculate total cost-effectiveness ratios as this has been recommended for LMICs [41]. Standard procedures will be followed for imputation of missing values as well as for the analysis of uncertainty and non-normal distributions. Further statistical analysis of costs will be performed to test for statistical significance of results, while bootstrapping will be performed to estimate variability in key parameters. Relevant deterministic and probabilistic sensitivity analyses will be conducted to understand the robustness of the results and the impact of each parameter on the model’s results. The robustness of each treatment will be shown through 95% confidence intervals around the cost-effectiveness ratio, as well as with for the sensitivity analyses (i.e., discounting of 3%). We will generate a cost effectiveness acceptability curve to summarize the uncertainty in the estimates of the cost-effectiveness analyses [42].

#### Process evaluation

Process evaluation will be guided by the Consolidated Framework for Implementation Research (CFIR) that will inform formative evaluation and help to build the implementation knowledge base for proposed interventions across study settings [43]. We will purposively sample up-to 15 patient-carer dyads from each treatment arm (total 30 patient-carer dyads) ensuring maximum variation and diversity (age, gender, socioeconomic status, and geographical location) and up to 15 patient-carer dyads who ‘drop out’ before completion (total 30 patient-carer dyads). Based on our previous experience [44] and published evidence [45], we are confident that we will achieve data saturation [46] with these numbers of interviews. We will interview all trial therapists for both interventions to explore experiences of delivering interventions, barriers and facilitators, the content and phrasing of discussions with patients and carers, and their views about approaches that work best and least. Transcripts will be analyzed using the Framework analysis [47] incorporating both inductive and deductive coding. The deductive coding will be informed by the CFIR with inductive coding ensuring no relevant data is lost.

### Ethical considerations

The authors assert that all procedures contributing to this work comply with the ethical standards of the relevant national and institutional committees on human experimentation and with the Helsinki Declaration of 1975, as revised in 2008. All procedures involving human subjects/patients were approved by the National Bioethics Committee of Pakistan (Ref: No.4-87/NBC-905/23/1199) and the Centre for Addiction and Mental Health (CAMH) Research Ethics Board, Toronto, Canada (REB # 2023/017). All members of the research team will comply with the International Conference on Harmonization Good Clinical Practice (ICH-GCP) Guideline. Research staff will be trained in good clinical practice (GCP) and will not begin data collection until the GCP certification is successfully completed. All information provided by the participants will be kept confidential and authorization will be required prior to accessing data. Patient-identifying information will not be published. Privacy and confidentiality of participants will be maintained. Participation in the trial will be voluntary and participants will have the right to withdraw from the study at any time, for any reason. Withdrawal from the trial will have no effect on routine care.

#### Supervision

Regular training and continuous supervision will not only facilitate the therapists to deliver interventions more effectively but will also expand and refine their skills. Regular training and supervision will also help to maintain and improve the fidelity of interventions, which is fundamental to the validity of the study. Fidelity and adherence to the manuals of the interventions will be assessed via recorded sessions using Cognitive Behaviour Therapy Rating Scale (CTRS) and the Cognitive Therapy for Psychosis Adherence Scale (CTPAS) [41, 42]. Ten percent of recorded sessions will be reviewed internally by co-Investigators. These steps will serve to ensure fidelity but also adherence to the fundamental components of both interventions.

#### Adherence

Adherence measures for patient and family/carer participants will include the Psychosocial Treatment Compliance Scale (PTCS) [41] and The Treatment Adherence Rating Scale (TARS) [42]. We will also document the number of intervention sessions attended.

### Safety

There are no anticipated risks to the participants. Anyone identified with acute psychosis or at acute risk of suicide will be assisted to immediately contact local mental health professionals. If required, the research team will accompany the participant to the local mental health service provider. We will provide written protocols including risk management and safeguarding. All assessments will be completed in accessible, private, and appropriate venues that suit the needs and preferences of participants. The appointments will be scheduled at times convenient to participants, considering education, household commitments (especially for women) and employment commitments. Participants will not be exposed to a risk of physical and mental harm that is greater than that typically encountered in normal life and the recruitment materials will direct participants to relevant supports if participation raises any concerns. All serious adverse events (SAEs) will be reported to the chief investigator and the Trial Steering Committee (TSC). All SAEs of a related and unexpected nature will be reported to the main Research Ethics Committee (REC).

### Study management and monitoring

This large RCT requires effective on-going managerial and scientific coordination by an experienced trial manager. The trial manager will report to the Principal Investigator (PI) and provide reports to the Trial Steering Committee (TSC). The TSC will include the PI, patient representative, an independent statistician, and an independent chair. The TSC will meet annually, but twice in the first year; with advice from the chair as needed between meetings. A Data Safety and Monitoring Committee (DSMC) will meet annually to monitor the data and advise the TSC on any ethical or safety reasons for why the trial should not continue. If there are data safety or efficacy issues the DSMC may determine the modification or deliberate project continuation and make recommendations to the TSC.

## Discussion

The World Bank suggests that effective treatment of FEP is likely to deliver the greatest economic and social impact in low-resource settings, reducing disability and enhancing productivity. The burden of mental disorders in Pakistan was estimated at $4 billion in 2006 [48]. Over 60% of Pakistan’s population is under 30 years of age and 29% are aged between 15 and 29 years [49], the typical age range at which young people first experience psychosis. Young people in LMICs are especially vulnerable to developing severe mental disorders like psychosis [50, 51]. Increased vulnerability reflects high rates of poverty, violence, political instability, trauma, stigma, cultural beliefs, and humanitarian crises. Economic growth and stability are top national priorities for Pakistan and addressing the burden of mental disorders including psychosis is a challenge to this emerging economy. Antipsychotics are the mainstay treatment, however, 25–50% of patients with psychosis continue to experience distressing symptoms despite compliance with medication [52]. International treatment guidelines [5–7] endorse CBT and FI for all individuals with early psychosis, however, evidence for their efficacy from low-resource settings, including Pakistan, is scarce. CBT requires the participant to be actively engaged, however, there are many individuals with FEP who may not want to directly engage in psychosocial interventions. FI, on the other hand, can improve patient outcomes without the patient actively participating in the intervention. Our published pilot feasibility studies demonstrate that such interventions are acceptable, feasible and may lead to improvement in clinical outcomes for Pakistani patients with psychosis. Building on these pilot studies, to our knowledge this will be the first large-scale multi-centre RCT confirming clinical efficacy and cost-effectiveness for these interventions, a critical next step to support subsequent implementation and scale up. Limited research exists directly comparing these two treatment options for psychosis, while each has been shown to independently improve outcomes [8, 53]. This trial has the potential to inform the implementation of evidence-based and scalable interventions in low-resource settings in LMICs and high-income countries.

## Supplementary Information

**Additional file 1.**

## Data Availability

The datasets used and/or analysed during the current study are available from the corresponding author on reasonable request.
