# Supplementary material for: Culturally adapted psychosocial interventions (CaPSI) for early psychosis in a low-resource setting: study protocol for a large multi-center RCT
Source: BMC Psychiatry. 2023 Jun 16;23:444. doi: 10.1186/s12888-023-04904-8 (PMC10276384; doi:10.1186/s12888-023-04904-8)
Supplement: Supplementary file 1 — Additional file 1. [file 12888_2023_4904_MOESM1_ESM.docx]

## **Appendix 1**

### Eligibility criteria

**Inclusion and exclusion criteria**

Patient participant inclusion criteria:

- Individuals of all genders aged over 18 years; diagnosis of schizophrenia confirmed by Structured Clinical Interview for DSM (SCID) meeting DSM-5 criteria for schizophrenia, schizophreniform or schizoaffective disorder
- scored at least 4 on the PANSS delusions or hallucinations items, or at least 5 on suspiciousness, persecution, or grandiosity items
- stable on medication for the past four weeks
- in contact with mental health services
- within 3 years of diagnosis
- able to demonstrate the capacity to provide informed consent to take part in the study
- potential participants must have a carer or relative who is also willing to participate in the study to be eligible

Patient participant exclusion criteria:

- Active DSM-5 substance use disorder (except nicotine or caffeine) or dependence within the last three months
- A score of 5 or more on the PANSS conceptual disorganisation item
- Individuals who have received structured psychological intervention within the past 3 months
- Relevant CNS or other medical disorders that would impact participation
- Diagnosis of intellectual disability
- Unstable residential arrangements

Family member/Carer participant inclusion criteria:

- Living with or spending at least 10 hours per week in face-to-face contact with an individual with early psychosis and assuming a caring role
- Age>18 years
- Able to give informed written consent.

Family member/Carer participant exclusion criteria:

- Active DSM-5 substance use disorder
- Received psychological intervention within the past 3 months
- Unstable residential arrangements.

## **Appendix 2**

## **Primary outcomes:** Positive and Negative Syndrome Scale (PANSS) ^(1)^ Total score: Reduction in symptom severity measured using PANSS total score post intervention (three months), six months and twelve months post intervention.

## **Secondary outcomes:**

## **Patients:**

## PANSS Positive Subscale

## PANSS Negative Subscale

## PANSS General Psychopathology Subscale

## Depressive symptoms as measured by the Calgary Depression Scale for Schizophrenia (CDSS) ^(2)^

## Health-related quality of life measured using EQ-5D^(3)^

## Measure of disability using WHODAS^[48]^

## Measure of insight with the Schedule of Assessment of Insight^(4)^.

## **Family/carer:**

## Experience of Caregiving Inventory (ECI) ^(5)^

## Carer Well-Being and Support (CWS) ^(6)^

## Illness Perception Questionnaire (IPQ) ^(7)^

## Generalized Anxiety Disorder (GAD-7; ^(8)^)

## Patient Health Questionnaire (PHQ-9; ^(9)^).

## **Appendix 3**

## **Schedule of assessments**

| **INSTRUMENT FOR PATIENTS** | **TIMEPOINT** | | | |
| --- | --- | --- | --- | --- |
|  | Baseline | 3 months | 6 months | 12 months |
| Demographic questionnaire | X |  |  |  |
| SCID for the DSM-5 | X |  |  |  |
| Positive and Negative Syndrome Scale (PANSS) | X | X | X | X |
| Calgary Depression Scale for Schizophrenia (CDSS) | X | X | X | X |
| EuroQol-5D (EQ- 5D) | X | X | X | X |
| The Self-report World Health Organization - Disability Assessment Schedule | X | X | X | X |
| Schedule of Assessment of Insight (SAI) | X | X | X | X |
| **INSTRUMENT FOR CARERS** | **TIMEPOINT** | | | |
|  | Baseline | 3 months | 6 months | 12 months |
| Demographic questionnaire | X |  |  |  |
| SCID for the DSM-5 | X |  |  |  |
| Experience of Caregiving Inventory (ECI) | X | X | X | X |
| Carer Well-Being and Support (CWS) | X | X | X | X |
| Illness Perception Questionnaire (IPQ) | X | X | X | X |
| Generalized Anxiety Disorder (GAD-7) | X | X | X | X |
| Patient Health Questionnaire (PHQ-9) | X | X | X | X |

**References:**

1. Kay SR, Fiszbein A, Opler LA. The positive and negative syndrome scale (PANSS) for schizophrenia. Schizophr Bull. 1987;13(2):261-76.

2. Addington D, Addington J, Maticka-Tyndale E. Assessing depression in schizophrenia: the Calgary Depression Scale. Br J Psychiatry Suppl. 1993(22):39-44.

3. Herdman M, Gudex C, Lloyd A, Janssen M, Kind P, Parkin D, et al. Development and preliminary testing of the new five-level version of EQ-5D (EQ-5D-5L). Qual Life Res. 2011;20(10):1727-36.

4. Ustun TB, Chatterji S, Kostanjsek N, Rehm J, Kennedy C, Epping-Jordan J, et al. Developing the World Health Organization Disability Assessment Schedule 2.0. Bull World Health Organ. 2010;88(11):815-23.

5. Szmukler GI, Burgess P, Herrman H, Benson A, Colusa S, Bloch S. Caring for relatives with serious mental illness: the development of the Experience of Caregiving Inventory. Soc Psychiatry Psychiatr Epidemiol. 1996;31(3-4):137-48.

6. Quirk AS, S.; Hamilton, S.; Lamping, D.; Lelliott, P.; Stahl, D., et al. . Development of the carer well‐being and support (CWS) questionnaire. Mental Health Review Journal. 2012;17(3):128-38.

7. Broadbent E, Petrie KJ, Main J, Weinman J. The brief illness perception questionnaire. J Psychosom Res. 2006;60(6):631-7.

8. Spitzer RL, Kroenke K, Williams JB, Lowe B. A brief measure for assessing generalized anxiety disorder: the GAD-7. Arch Intern Med. 2006;166(10):1092-7.

9. Lowe B, Kroenke K, Herzog W, Grafe K. Measuring depression outcome with a brief self-report instrument: sensitivity to change of the Patient Health Questionnaire (PHQ-9). J Affect Disord. 2004;81(1):61-6.
